# Brief Review of the Role of Glycogen Synthase Kinase-3*β* in Amyotrophic Lateral Sclerosis

**DOI:** 10.1155/2011/205761

**Published:** 2011-03-20

**Authors:** Seong-Ho Koh, Wonki Baek, Seung H. Kim

**Affiliations:** Department of Neurology, College of Medicine Hanyang University, no. 17 Haengdang-Dong, Seongdong-Gu, Seoul 133-792, Republic of Korea

## Abstract

Glycogen synthase kinase-3*β* (GSK-3*β*) is known to affect a diverse range of biological functions controlling gene expression, cellular architecture, and apoptosis. GSK-3*β* has recently been identified as one of the important pathogenic mechanisms in motor neuronal death related to amyotrophic lateral sclerosis (ALS). Therefore, the development of methods to control GSK-3*β* could be helpful in postponing the symptom progression of ALS. Here we discuss the known roles of GSK-3*β* in motor neuronal cell death in ALS and the possibility of employing GSK-3*β* modulators as a new therapeutic strategy.

## 1. Introduction

Amyotrophic lateral sclerosis (ALS), characterized by progressive muscle wasting and weakness, is a progressive, fatal neurodegenerative disorder characterized by the preferential loss of motoneurons. The precise pathogenic mechanisms on motoneuron death need to be clearly elucidated, although several theories, including oxidative stress, glutamate toxicity, calcium-mediated toxicity, neurotrophic factor withdrawal, genetic defects, immune-inflammation, and accumulation of abnormal proteins, have been proposed as pathogenic mechanisms of familial/sporadic ALS [1–8]. However, recent discoveries of copper-zinc superoxide dismutase (SOD1) mutant genes, which cause familial ALS, have significantly contributed to the current understanding of the pathogenic mechanisms of ALS [1, 4–8].

Glycogen synthase kinase-3 (GSK-3), originally identified as a regulator of glycogen synthesis [9], is now known to be a multifaceted enzyme affecting a diverse range of biological functions from gene expression to cellular architecture and apoptosis [10]. Two closely related isoforms of GSK-3, GSK-3*α*, and GSK-3*β*, exist in mammals [11]. GSK-3*β* has more important roles than GSK-3*α* in the nervous system. Moreover, the role of GSK-3*β* has been identified as one of the important enzymes regulating pathogenic mechanisms of Alzheimer's disease (AD) and Parkinson's disease (PD). The abnormal increase in the level and activity of GSK-3*β* has been associated with neuronal death, paired helical filament tau formation, and neurite retraction in AD and in GSK-3*β* mediated 6-hydroxydopamine-induced neuronal death in *in vitro* and *in vivo* models of PD [12–14]. Recently, it has been reported that the role of GSK-3*β* is important in the pathogenic mechanisms of ALS. For example, GSK-3*β* is increased in the thoracic spinal cord tissue of patients with sporadic ALS and in motoneurons transfected with G93A or the A4V mutant hSOD1 gene [13, 15]. 

In this review, we will focus our discussion on the following topics: (1) a description of GSK-3*β*, (2) the role of GSK-3*β* in neuronal cell death, (3) the role of GSK-3*β* in ALS, and (4) the development of new GSK-3*β* inhibitors.

## 2. A Description of GSK-3*β*

GSK-3 is a serine/threonine protein kinase and a key enzyme involved in glycogen metabolism [16]. Activated GSK-3 phosphorylates and inactivates glycogen synthase, converting glucose to glycogen. In mammals, GSK-3 is encoded by distinct two genes, *GSK-3α* and *GSK-3*β** [11]. GSK-3*α* and GSK-3*β* are constitutively active and usually phosphorylate substrates that are prephosphorylated. Especially focused on GSK-3*β*, many studies have shown that GSK-3*β* is activated by phosphorylation of the tyrosine 216 residue (Tyr216) located in the kinase domain and inactivated by phosphorylation of the amino-terminal serine 9 residue (Ser9) and that GSK-3*β* participates in a variety of cellular processes, including the insulin and Wnt/wingless signaling pathways, and in the regulation of metabolic, structural, and signaling protein functions, such as activator protein-1, cyclic AMP response element binding protein, the nuclear factor of activated T cells, heat shock factor-1, and *β*-catenin [10, 16–20]. GSK-3*β* is also known to directly affect the CCAAT/enhancer binding protein, Myc; the heat shock transcription factor-1 (HSTF-1), tau; nuclear factor *κ*B (NF*κ*B); p53; caspase-3; the release of cytochrome *c* [15–20]. 

The activity of GSK-3*β* is negatively regulated by insulin. Specifically, insulin enhances the activation of phosphatidylinositol 3-kinase (PI3K), which in turn increases the expression of antiapoptotic proteins and inhibits the activity of proapoptotic proteins. PI3K activates the downstream target Akt/protein kinase B by phosphorylating it. Phosphorylated Akt (pAkt) subsequently phosphorylates and inhibits GSK-3*β*. When excessive stress occurs, however, cell death is induced and activates the phosphatase and tensin homolog (PTEN), a 3′-phosphatase that converts PI(3,4)P_2_ to PI(4)P and PI(3,4,5)P_3_ to PI(4,5)P_2_, in cells, and activated PTEN then inactivates PI3K via the suppression of PI(3,4,5)P_3_. Inactivated PI3K cannot phosphorylate Akt, and the decrease in pAkt reduces phosphorylation of GSK-3*β*, all of which causes an increase in the active form of GSK-3*β* [15–20]. Activated GSK-3*β* inhibits HSTF-1, resulting in the mitochondrial death pathway and the release of cytochrome *c* from mitochondria. Finally, released cytochrome *c *induces apoptosis by activating caspase-9 and caspase-3 [21–23]. GSK-3*β* is also involved in the Wnt signaling pathway, which also inhibits GSK-3*β* activity and the phosphorylation and degradation of substrates by GSK-3*β* [24, 25]. 

Regarding GSK-3*β* localization, GSK-3*β* has traditionally been classified as a predominantly cytosolic enzyme [26]; however, it was recently shown that smaller but much more active pools of GSK-3*β* are present in the nucleus and mitochondria [18].

## 3. The Role of GSK-3*β* in Neuronal Cell Death

As described above, there are two isoforms of GSK-3 in mammals, GSK-3*α* and GSK-3*β*. GSK-3*β* is more prominent in the nervous system and has been reported to play an important role in neuronal cell death [15, 19, 27], although it has recently been proposed that GSK-3*α* also plays a role in this process [28]. GSK-3*β* has been identified as one of the principal enzymes involved in the pathogenic mechanisms of neurodegenerative disorders and ischemic stroke. 

There are numerous studies showing that abnormal increases in the level and activity of GSK-3*β* induce neuronal cell death paired helical filament tau formation, and neurite retraction in Alzheimer's disease (AD) [14]. In particular, it is commonly accepted that amyloid beta protein activates GSK-3*β*, and activated GSK-3*β* mediates phosphorylation of tau protein, induces neuronal cell death, and disrupts axonal transport through an NMDA receptor-dependent mechanism [29].

GSK-3*β* is also known to participate in the pathogenesis of PD [12]. *α*-Synuclein protein deposition into Lewy bodies is a pathologic marker of PD, and *α*-Synuclein induces neurotoxicity. In addition, GSK-3*β* is thought to be important in *α*-Synuclein-induced neurotoxicity. It has been reported that *α*-Synuclein activates GSK-3*β*, enabling the hyperphosphorylation of tau proteins; however, the precise mechanism by which *α*-Synuclein contributes to the activation of GSK-3*β* remains to be elucidated [30]. 

In stroke, GSK-3*β* has been reported to be involved in neuronal cell death, whereas treatment with GSK-3*β* inhibitors in the acute state reduces infarction volume and improves neurobehavioral function [31, 32]. In the chronic state, GSK-3*β* inhibitors promote neurovascular remodeling after stroke and improve postischemic stroke sequelae [33, 34].

## 4. The Role of GSK-3*β* in ALS?

Recent studies have reported that an abnormal increase in GSK-3*β* is found in *in vitro* and *in vivo* models of ALS, in the thoracic spinal cord tissue of patients with sporadic ALS [13], and in the frontal and temporal cortices of ALS patients. Moreover, its suppression attenuates disease progression in an ALS mouse model [35–37]. Specifically, Hu et al. reported that protein kinases, such as GSK-3*β*, are increased in the thoracic spinal cord tissue of patients with sporadic ALS [13]. Yang et al. reported that the expression of GSK-3*β* and phospho-beta-catenin, which reflects the activity of GSK-3*β*, was increased in ALS patients compared to that of controls and that GSK-3*β* immunoreactive neurons were mainly located in layer II and layer III of the frontal cortex and in layer II of the hippocampus [35]. In our previous findings, both GSK-3*β* expression and activity were increased in motor neurons transfected with the G93A, or A4V mutant hSOD1 genes and in motor neurons transfected with the G93A or A4V mutant hSOD1 genes [15, 38]. Taken together, these findings support the potential role for GSK-3*β* as a therapeutic target in ALS. 

There have been many studies showing that GSK-3*β* inhibition can suppress disease progression of ALS in both *in vitro* and *in vivo* models. We have reported that GSK-3*β* inhibition, using a specific inhibitor or using materials with GSK-3*β* inhibitory effects, reduced motor neuronal cell death in motor neurons transfected with the G93A or A4V mutant hSOD1 genes in an *in vitro* model of ALS [15, 39]. Recently, Calderό et al. also reported that lithium, a GSK-3*β* inhibitor, prevents the excitotoxic cell death of motor neurons [40]. Feng et al. and our group also reported that disease onset, disease progression, and survival in ALS mouse models were prolonged by the treatments of specific inhibitors of GSK-3*β* or materials with GSK-3*β* inhibitor effects. The protective mechanisms of GSK-3*β* inhibition in *in vitro* and *in vivo* models of ALS are as follows. GSK-3*β* inhibition decreases motoneuronal cell death by restoring survival-related signals, such as HSTF-1; by reducing death-related signals, such as cytochrome *c*, thereby reflecting mitochondrial injury, activated caspase-3, and cleaved forms of poly (ADP-ribose) polymerase; by decreasing inflammation-related signals such as cyclooxygenase-2 and intercellular adhesion molecule 2. 

Why and how GSK-3*β* is activated, however, remain to be elucidated. Based on previous reports, including studies from our laboratory, we hypothesize that the disturbance of signaling proteins upstream of GSK-3*β*, which control the activity or the expression of GSK-3*β*, might cause overactivation or overexpression of GSK-3*β*. For example, a decrease in or the abnormality of proteins involved in the PI3K pathway, such as PI3K and Akt, might be associated with overactivation of GSK-3*β* in ALS, considering the many reports showing that a specific PI3K activator or materials with a PI3K-activating effect modify GSK-3*β* activity and reduce motoneuronal cell death [41–46]. There was, however, a report showing that changes in the PI3K/Akt survival pathway were not found in spinal cord motor neurons in a mouse model of familial ALS [47]. Clear answers to these issues should be elucidated in future studies and could be helpful for the development of new and effective therapeutic strategies for ALS.

## 5. The Development of New GSK-3*β* Inhibitors

Considering that GSK-3*β* is overactivated in ALS and that it plays an important role in motoneuronal cell death, the development of new GSK-3*β* inhibitors might be helpful in the treatment of ALS, so there are many new GSK-3*β* inhibitors under development (Table 1) [48]. In the development of new GSK-3*β* inhibitors, it should be considered that overinhibition of GSK-3*β* activity can conversely cause neuronal cell death because of GSK-3*β*'s role in a variety of cellular processes such as glucose metabolism. We previously showed that treatment with higher concentrations of GSK-3*β* inhibitors, above the optimal concentrations that prevent neuronal cell death by oxidative stress, induced neuronal cell death [49].

Another consideration is that chronic inhibition of GSK-3*β* could increase several transcription factors and *β*-catenin, which could contribute to the development of cancer. In detail, *β*-catenin binds to transcription factors and to other proteins, such as axin, a component of the Wnt signaling pathway. Active GSK-3*β* phosphorylates *β*-catenin and is involved in the degradation of the *β*-catenin complex. Chronic inhibition of GSK-3*β*, however, increases *β*-catenin and other proteins, such as adenomatous polyposis coli (APC), and this process may lead to higher incidences of cancer [24, 25]. 

Taken together, newly developed GSK-3*β* inhibitors for the treatment of ALS should have an optimal and balanced inhibitory effect on GSK-3*β*, and the appropriate dose needs to be carefully examined. Furthermore, it is extremely desirable to identify the GSK-3*β* inhibitor dose that results in no effect on *β*-catenin degradation, to prevent cancer formation.

## 6. Conclusions

GSK-3*β* plays extremely important roles in the motoneuronal cell death related to ALS, and the development of specific GSK-3 inhibitors might be an effective strategy for the treatment of ALS.

## Figures and Tables

**Table 1:** New GSK-3*β* inhibitors under development.

Inhibitor	Class	IC50 (mM)	
		GSK-3*α*	GSK-3*β*
ARA014418	Thiazole	—	0.1040
CHIR98014	Aminopyrimidine	0.0007	0.0006
CHIR99021 (CT99021)	Aminopyrimidine	0.0100	0.0070
Compound 17	Chloromethyl thienyl ketone	—	1.0000
Compound 1A	Pyrazolopyrimidine	—	0.0160
Compound 29	Azaindolylmaleimide	—	0.0340
Compound 46	Azaindolylmaleimide	—	0.0360
Compound 5a	Bisindolylmaleimide	—	0.0180
Compound 8b	Triazole	—	0.2800
CT20026	Aminopyridine	0.0040	0.0040
I5	Anilinoarylmaleimide	0.0760	0.1600
SB216763	Arylindolemaleimide	0.0340	0.0750
SB415286	Anilinomaleimide	0.0780	0.1300

GSK-3: glycogen synthase kinase.
